# Quantification of effective orbital volume and its association with axial length of the eye. A 3D-MRI study

**Published:** 2019

**Authors:** Georgios Bontzos, Efrosini Papadaki, Michael Mazonakis, G. Thomas Maris, Zoi Kapsala, Styliani Blazaki, E. Eleni Drakonaki, T. Efstathios Detorakis

**Affiliations:** * Department of Ophthalmology, University Hospital of Heraklion, Crete, Greece; ** Department of Radiology, University Hospital of Heraklion, Heraklion, Greece; *** Department of Radiology, University Hospital of Heraklion, Heraklion, Greece; **** Independent Imaging Services, Heraklion, Crete, Greece

**Keywords:** orbit, axial length, magnetic resonance tomography, orbital volume

## Abstract

**Objective:** To measure the effective orbital volume (EOV) from magnetic resonance images, and investigate its relationship with axial length (AL) in those parameters.

**Methods:** Cross-sectional, 3D-MRI study. 54 eyes of 54 patients (25 males) were included in this work. Patient weight, height and head circumference were also measured. Orbital and eyeball volumes were calculated after image segmentation. The difference between those values volume was assessed, estimating the EOV for each eye.

**Results:** Mean eyeball volume was 7.83 ± 2.27 mm3, mean orbital volume 26.81 ± 0.59 mm3 and EOV 21.64 ± 0.19 mm3. The orbital volume was significantly higher in the male group (Wilcoxon signed-rank tests Z=-1.51, p<0.001; Z=-3.57, p<0.001 respectively). EOV was significantly correlated with AL in both males (r=-0.71, p<0.001) and females (r=-0.73, p<0.001), whereas it was also significantly associated with patient height (r=0.261, p=0.03). Associations between EOV and other age, axial and anthropometric characteristics were not statistically significant.

**Conclusions:** Findings of this study could be of valuable importance in various clinical situations in which quantification of orbital volume is needed, such as orbital decompression in Graves’ orbitopathy, volume restoration in orbital fractures or other orbital reconstructive surgery. In surgical interventions, clinical relationships should be carefully taken under consideration to avoid iatrogenic injury.

**Abbreviations:** EOV = Effective orbital volume, AL = Axial length, ROI = Region of interest

## Introduction

The orbit is a pear-shaped cavity, highly variable in size and volume among individuals. Anatomical alternations in orbital structure affect clinical decisions and surgical outcomes [**1**]. Both osseous and soft tissue of the orbit display significant differences in healthy population. Previous studies have explored those alternations concerning the size of the orbit and the eyeball, and the position of the latter in the former [**2**]. The effective orbital volume (EOV) is defined as the difference between the eyeball volume and the orbital cavity volume [**3**]. It provides an estimation of the available space in the orbital cavity to accommodate the eyeball.

Furthermore, pathological sequelae of various diseases can manifest as structural deficits or distortion of the orbit. Examples are intra-orbital tumors [**4**], inflammatory disorders [**5**], congenital diseases, and traumatic skull fractures [**6**]. Changes in the orbital volume in these conditions are usually observed as signs of exophthalmos, enophthalmos, and dystopia. Quantification of the orbital cavity and the soft tissue structures is helpful in the design of surgical interventions in orbital disease as well as in the understanding of the pathogenesis and clinical course in various conditions [**7**,**8**]. Importantly, it has been suggested that EOV may be used to determine intra-orbital eyeball position [**3**], especially in cases with a “congested” orbit, i.e. when the available space within the orbit may be limited forcing the eyeball to protrude forward [**3**].

Advances in radiological techniques of computed tomography (CT) and three-dimensional magnetic resonance tomography (3D-MRI) have enabled detailed imaging of the orbit and its associated structures. However, in literature, there are few articles [**3**,**9**,**10**], which have tried to quantify the anatomical variations of the orbit and its dynamic relationship with the eyeball using MRI, with respect to demographic and anthropometric parameters. 

The aim of the present 3D-MRI study was to: (a) quantify the orbital volume and its relationship with the eyeball by measuring EOV and (b) explore the association between EOV and axial length (AL) of the eye as well as anthropometric parameters.

## Methods

This was a single center, cross-sectional study at the University Hospital of Heraklion, Crete, Greece. For the purpose of this work, 54 Caucasian patients (25 males, 46.2%) with a mean age 57.78 ± 14.71 years (range 23-82) were included. The study was approved by local ethics committee and adhered to the tenets of the Declaration of Helsinki. The purpose of this study was explained to all participants who gave a signed written consent. Height, weight, and head circumference measurements were taken. The head circumference was measured around the most projecting part of the frontal bone and the most projecting part of the occipital bone, using a standardized procedure. AL in all eyes was measured using the IOL Master 500 (Carl Zeiss Meditec, Jena, Germany). 

For each patient, 3D-MRI was performed. MR imaging was performed using a clinical 1.5T whole-body superconducting imaging system (MAGNETOM Sonata/ Vision, Siemens Healthcare, Erlangen, Germany), equipped with high performance gradients (Gradient strength: 40 mT/ m, Slew rate: 200 mT/ m /ms) utilizing a standard circular polarized (CP) body coil as a transmitter and a linear polarized (LP) head coil as a receiver. The comprehensive MR imaging protocol consisted of one 3D T1w sequence [(3D-VIBE) (Volume Interpolated Breath hold Examination)] and one 3D (T2/ T1) w sequence [(3D-CISS) (Constructive Interference on the Steady State). The participants were instructed to keep both eyes closed with minimal movement during the scanning. The image resolution voxel were: (0.1 x 0.1) mm2 in- plane spatial resolution and 0.625 mm cross-plane spatial resolution (slice thickness). Axial, coronal, and sagittal images were obtained. The typical scanning time for each participant was approximately 11 minutes.

For the current work, only the right eye was analyzed. DICOM images were exported to the open source imaging processing software 3D Slicer v.4.7.0, for image segmentation, by applying manual 3D volume rendering. The eyeball volume and the orbital volume were calculated in axial slices. For volume calculations, the region of interest (ROI) in each slice was multiplied by slice thickness, and the whole volume was determined after summation of the volume of each slice. The orbit boundaries were manually delineated in axial slices. The posterior border was defined as the crossing line of the medial and lateral walls of the optic foramen, and the anterior border was defined as the connecting line between the medial and lateral orbital walls. Difference between the orbital and the eyeball volume was assessed, estimating the EOV for each eye. Furthermore, we calculated the intercanthal distance using the axis connecting the bone rims of the outer canthi (intercanthal line). All measurements were conducted by two experienced professionals specialized in head and neck anatomy.

Statistical analysis was performed using SPSS (IBM SPSS Statistics for Windows, Version 22.0; Armonk, NY, USA). Descriptive statistics are presented as mean ± SD. All p values relate to two-sided tests with a significance level of a = 0.05. Graphical displays were illustrated using GraphPad Prism (Graphpad Software Inc, San Diego, CA). The power of the study, evaluated by G*power (version 3.1.9.2, Universitat Kiel, Germany) was 0.606 for the Pearson bivariate correlation coefficient given an effect size (r) of 0.3 and an error of 0.05. A two-step cluster analysis was applied to the data set in order to determine whether it could classify each participant into different characteristic groups based on gender, age, and AL. These measures were selected in order to reduce the effects of co-variance, as they are measures that are independent of one another.

## Results

Patients had a mean axial length of 25.83 ± 4.46 mm (range: 16.6mm–36.02mm). Cluster analysis indicated two clusters with a size ratio of 1.16 (“good separation” based on Akaike’s information criterion, AIC). Cluster 1 (29 orbits, 100% male, mean age=55.9, mean AL=25.73) and cluster 2 (25 orbits, 100% females, mean age=60.16, mean AL=25.82). 

Mean eyeball volume was 7.83 ± 2.27 mm3 (range: 2.69-11.38, 8.01 ± 0.44 mm3 for males and 7.67 ± 0.43 mm3 for females) and displayed a significant correlation with AL (r=0.954, p=0.001). Total orbital volume was 26.81 ± 0.59 mm3 (range: 19.92–32.71, 27.62 ± 0.38 mm3 for males and 25.34 ± 0.31 mm3 for females) and was not significantly correlated with AL (r=0.348, p=0.547). EOV was calculated with mean value of 21.64 ± 0.19 (range: 18.98-24.66). The male group had mean EOV=21.93 mm3 while the female group had EOV=20.28 mm3. Wilcoxon signed-rank tests were performed to compare the mean values between the two clusters. The tests indicated statistically higher orbital volume in the male group (Z=-1.51, p<0.001) and statistical significant higher EOV in the male group. (Z=-3.57, p<0.001).

**Fig. 1:**
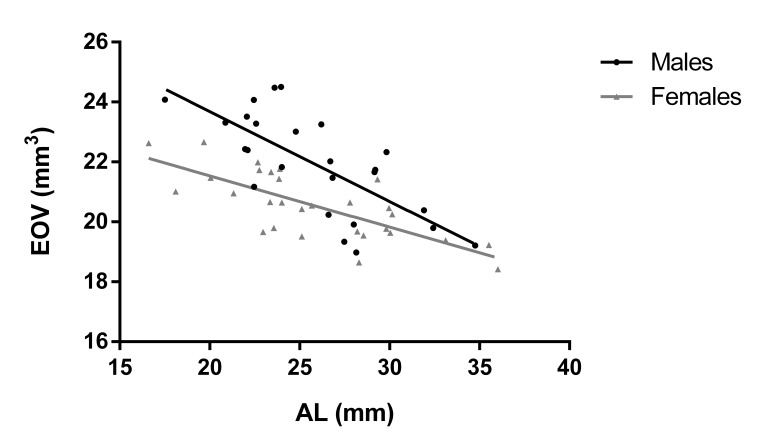
Scatterplot illustrating AL vs. EOV. Solid lines denote the lines of equality

Further measurements included anthropometric variables: height, weight, head circumference, and intercanthal distance. Results and comparisons between men and women are shown in **Table 1**. Correlation coefficients between orbital volume, ocular and anthropometric variables are presented in **Table 2**. Correlation coefficients for EOV are also presented in **Table 3**. EOV displayed a significant correlation with AL in our pool sample (r=-0.594, p<0.001), in males (r=-0.71,p<0.001) and females (r=-0.73 ,p<0.001). Data are illustrated in **Fig. 1**.

**Table 1:** Anthropometric measurements by Gender

		Gender	
	Male (n=25)	Female (n=29)	P*
Height (cm)	177.3 ± 7.8	163.1 ± 6.2	<0.001
Weight (kg)	84.8 ± 8.5	75 ± 6.3	0.023
Head circumference (cm)	58.9 ± 1.9	56.2 ± 2.4	0.015
Intercanthal distance (cm)	9.8 ± 0.9	9.3 ± 0.5	0.21
*Wilcoxon signed-rank test			

**Table 2:** Correlation coefficients (r) between Orbital Volume and measured variables

	r	P*
Age	-0.261	0.09
Axial length	0.199	0.282
Height	0.160	0.123
Weight	0.024	0.430
Head circumference	0.087	0.265
Intercanthal distance	0.279	0.098
*Pearson’s correlation coefficient		

**Table 3:** Correlation coefficients (r) between EOV and measured variables

	r	P*
Age	0.051	0.356
Axial length	-0.594	<0.001
Height	0.261	0.028
Weight	-0.044	0.377
Head circumference	0.201	0.072
Intercanthal distance	0.141	0.155
Orbital volume	0.08	0.282
*Pearson’s correlation coefficient		

## Discussion

In this study, we evaluated the anatomical differences of orbital structures in a healthy population with respect to ocular and anthropometric parameters. We also quantified EOV and investigated potential correlations of AL with EOV. Our sample included only Caucasians without orbital pathology. Results imply that EOV is significantly lower in the female group and that orbital volume is not significantly correlated with age, AL, and anthropometric characteristics. However, it was significantly higher in the male group. We also found a weak but significant correlation between EOV and patient height, and a significant negative association between EOV and AL, in both genders. 

Previous studies have explored the structure of the human orbits and its relationship with demographic and clinical parameters. Notable variability was found among different ethnic groups [**11**-**13**] and it was actually reported that orbital regions might display the greatest variation among facial measurements in both sexes [**14**]. Gender is another significant and independent factor of orbital and eyeball dimensions as reported in the literature [**15**-**17**], which also reflects the greater skull dimensions in men compared to women [**18**]. It has also been demonstrated that the bony elements of the orbit dramatically increase with age resulting in reduced orbital volume measurements [**19**]. Interestingly, studies have shown that volume reduction is more profound in women rather than in men, concurrently with hormonal changes during menopause [**20**,**21**]. Similarly, it was reported that eyeball volume of younger subjects was larger than of older adults [**22**]. In contrast, orbital fat volume was found to be increased with age [**23**,**24**]. Additionally, anthropometric measurements have been linked to orbital growth and it was found that height [**25**] and weight [**26**] display a positive correlation to orbital volume. The unique anatomy of the orbit and its relationships has been used for gender determination and identification in forensic medicine [**20**].

**Fig. 2:**
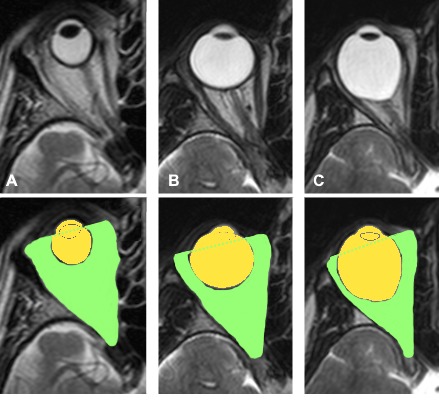
Examples of our patients showing a hyperopic (panel A: AL=16.6 mm), an emmetropic (panel B: AL=23.6 mm) and a highly myopic eye (panel C: AL=35.2 mm)

There are few published studies to evaluate the relative volume of the orbit and the eye globe. It has been proposed that the growth of the orbit is related to the growth of both the cranium and the eyeball and the orbital volume is proportional to eyeball volume in young children [**27**,**28**]. This assumption was also supported by Futura [**25**] and Bentley [**29**] who did not find sexual dimorphism in eyeball and orbital volume in adolescents. While several studies have reported that the eyeball volume is larger in myopic eyes than those of emmetropes [**30**,**31**], there has been no clear relationship between the orbital volume and AL of the eye [**32**,**33**]. In a previous study of our department, no significant correlation was found between AL and EOV in emmetropes [**3**]. However, there is indirect evidence that EOV is reduced in high myopia [**34**]. Strabismus fixus [**34**] and heavy eye phenomenon [**35**] can be attributed to mechanical restriction due to the limited available space of the orbital cavity, which forces the globe to dislocate from the muscle cone [**35**]. In this study, we assessed population varying from hyperopia to high myopia and explored the relationship between orbital and eyeball volume. We found that, with increasing AL, EOV appears to decrease. The clinical value of this finding can be observed in **Fig. 2**. In these 3 examples of hyperopia, emmetropia and high myopia we could visualize how there was limited free space available in the congested myopic orbital cavity. This finding might have important implications for the anatomical and functional status of the optic nerve, as previously suggested [**36**].

Findings of this study could also be beneficial in surgical planning. The orbit is an essential anatomical landmark of skull; its boundaries and soft tissue relationships need to be carefully measured before orbital and transorbital surgical interventions [**37**,**38**]. The orbital anatomy can be affected by various pathologies, such as fractures, tumors or following enucleation or evisceration. Reconstruction of the orbital cavity remains a challenge [**39**] especially for larger defects [**40**]. Complications of orbital repair include diplopia, enophthalmos, and infraorbital nerve hypoesthesia [**41**,**42**]. Awareness of the individual’s orbital morphology is crucial for surgical decisions. In particular, EOV calculations should be taken into consideration when attempting to restore the normal orbital relationships in cases of enophthalmos [**16**] and orbital decompression as well as in orbital implants procedures. Reduction in EOV is also useful in understanding disease pathogenesis and clinical signs of orbital protrusion or dystopia. The orbital volume can be affected by intraorbital tumors (e.g. retinoblastoma and rhabdomyosarcoma), inflammatory disorders, such as Graves’ disease and sarcoidosis or congenital diseases like Apert and Pfeiffer syndromes [**2**]. Quantification of the orbital tissues and EOV might also help understand the predisposition to certain orbital diseases between genders. For example, women are at greater risk of developing ocular motility problems associated with high myopia [**43**]. Limitation in this work is that it included only Caucasians. Therefore, results cannot be extended in different study populations.

To the best of our knowledge, this is the first study to measure the relative volume of the eyeball and the orbit, as expressed by EOV, in a wide range of AL. All measurements were performed using 3D-MRI. However, future studies should include both CT and MR images to investigate the reliability of calculations between modalities. 

**Funding**

This study has received funding by the General Secretariat for Research and Technology (GSRT) and the Hellenic Foundation for Research and Innovation (HFRI).

**Disclosures**

None.
